# ^64^Cu-SAR-Bombesin PET-CT Imaging in the Staging of Estrogen/Progesterone Receptor Positive, HER2 Negative Metastatic Breast Cancer Patients: Safety, Dosimetry and Feasibility in a Phase I Trial

**DOI:** 10.3390/ph15070772

**Published:** 2022-06-22

**Authors:** Keith Wong, Gemma Sheehan-Dare, Andrew Nguyen, Bao Ho, Victor Liu, Jonathan Lee, Lauren Brown, Rachel Dear, Lyn Chan, Shikha Sharma, Alessandra Malaroda, Isabelle Smith, Elgene Lim, Louise Emmett

**Affiliations:** 1 Department of Theranostics and Nuclear Medicine, St Vincent’s Hospital, 390 Victoria Street, Sydney, NSW 2010, Australia; 2 St Vincent’s Clinical School, University of New South Wales, Sydney, NSW 2010, Australia; 3 The Kinghorn Cancer Centre, St Vincent’s Hospital, Sydney, NSW 2010, Australia; 4 Garvan Institute of Medical Research, Sydney, NSW 2010, Australia

**Keywords:** breast cancer, bombesin, staging, PET, copper 64, SAR-BBN

## Abstract

Breast cancers are most frequently oestrogen receptor (ER) and progesterone receptor (PR) positive and [^18^F]Fluorodeoxyglucose PET-CT (FDG) has lower sensitivity for these subtypes. The gastrin-releasing peptide receptor (GRPR) is overexpressed in ER+/PR+ breast cancers. This study assessed the safety and potential of [^64^Cu]Cu-Sarcophagine (SAR)-Bombesin PET/CT (BBN) in re-staging metastatic ER+/PR+/human epidermal growth-factor-2-negative (HER2-) breast cancer. Seven patients with metastatic ER+/PR+/HER2- breast cancer undergoing staging underwent [^64^Cu]Cu-SAR-BBN PET-CT. Bloods, vital signs and electrocardiogram, blood tracer-clearance and dosimetry were undertaken. GRPR status was assessed in available metastatic biopsy samples. Staging with conventional imaging ([^18^F]FDG, bone scan and diagnostic CT) was within 3 weeks of [^64^Cu]Cu-SAR-BBN PET/CT. PET scans were assessed visually and quantitatively. Seven patients underwent imaging. One of the seven had de-novo metastatic breast cancer and six of the seven recurrent metastatic disease. Two of the seven had lobular subtype. No adverse events were reported. All seven patients were positive on conventional imaging (six of seven on FDG). [^64^Cu]Cu-SAR-BBN imaging was positive in five of the seven. Both [^64^Cu]Cu-SAR-BBN-negative patients had disease identified on [^18^F]FDG. One patient was [^64^Cu]Cu-SAR-BBN positive/[^18^F]FDG negative. Four of seven patients were [^64^Cu]Cu-SAR-BBN positive/[^18^F]FDG positive. In these four, mean SUVmax was higher for [^64^Cu]Cu-SAR-BBN than [^18^F]FDG (SUVmax 15 vs. 12). In the classical lobular subtype (two of seven), [^64^Cu]Cu-SAR-BBN was more avid compared to [^18^F]FDG (SUVmax 20 vs. 11, and 20 vs. <3). Dosimetry calculations estimated whole-body effective dose for 200 MBq of [^64^Cu]Cu-SAR-BBN to be 1.9 mSv. [^64^Cu]Cu-SAR-BBN PET/CT appears safe and may have diagnostic value in metastatic ER+/PR+/HER2- breast cancer, particularly the lobular subtype. Further evaluation is warranted.

## 1. Introduction

Metastatic breast cancer carries a poor prognosis with an overall five-year survival of 32% [1]. Detection of breast cancer metastases is essential for accurate staging and treatment planning. [^18^F]Fluorodeoxyglucose (FDG) PET-CT is now the standard of care for the staging of breast cancer in Australia. However, the heterogeneous nature of metastatic breast cancer means a targeted molecular tracer appropriate for one subtype of breast cancer may not be effective in another. Breast cancers that are oestrogen receptor (ER) positive, progesterone receptor (PR) positive and human epidermal growth factor receptor 2 (HER2) negative account for up to 80% of all breast cancers, and yet ER+/PR+ cancers have significantly lower avidity and sensitivity on FDG PET compared to triple-negative breast cancers (ER-/PR-/HER2-) [2,3,4].

The gastrin-releasing peptide receptor (GRPR) is overexpressed in breast cancer, particularly ER+/PR+ breast cancers and their metastases [5]. This makes GRPR a potential imaging and theranostic target in the diagnosis and treatment of metastatic breast cancer [5,6,7]. Bombesin is a tetra-decapeptide with high binding affinity to GRPR, and its uptake in breast cancer has been demonstrated in human trials [8,9].

Copper-64 is an alternative PET radioisotope and forms a chemically identical theranostic pair with Copper-67, a beta particle emitter. A GRPR antagonist conjugated to a sarcophagine derivative and radiolabelled with either copper-64 ([^64^Cu]Cu-SAR-BBN) or copper-67 ([^67^Cu]Cu-SAR-BBN) demonstrated high affinity for GRPR in animal studies [10,11]. This first human study aims to evaluate the safety and feasibility of [^64^Cu]Cu- SAR-BBN as a theranostic tool in women with metastatic ER+/PR+/HER2- breast cancer.

## 2. Results

### 2.1. Patient Characteristics

The patient characteristics are detailed in Table 1 and Table 2. In total, nine patients were enrolled between July 2020 and July 2021. All enrolled patients had proven ER+/PR+/HER2- breast cancer on histopathology prior to enrolment. Two of nine patients were enrolled but not imaged with [^64^Cu]Cu-SAR-BBN, due to clinical deterioration. Seven patients underwent all imaging, including [^64^Cu]Cu-SAR-BBN PET-CT. Median age was 60 (38–81), and six of seven patients had received previous lines of treatment, which included chemotherapy (three of seven), endocrine therapy (five of seven) and targeted therapy (six of seven). One of seven had newly diagnosed de-novo metastatic breast cancer. Two of seven patients had invasive lobular type and five of seven invasive ductal type on initial biopsy. Six of seven patients had metastatic disease in bone, four of seven in liver and four of seven in nodes on conventional imaging.

### 2.2. Safety and Adverse Events

No adverse events or change in vital signs or ECGs were reported. There were no changes to the haematological, biochemical or coagulation profiles between baseline and at 1 h post injection.

### 2.3. Dosimetry and Blood Clearance

The [^64^Cu]Cu-SAR-BBN PET-CT scans and blood clearance data were acquired at three time points for five of seven patients and at two time points for two patients (for one patient, the 24 h and for the other patient the 3 h scan and blood data were not acquired). Dosimetry calculations estimated the whole-body effective dose to be 0.0095 mSv/MBq or 1.9 mSv for a 200 MBq injection of [^64^Cu]Cu-SAR-BBN (Table 3). From all organs, the pancreas demonstrated the highest uptake but with good clearance over 24 h. Doses to critical organs, such as the bone marrow (mean ± SD: 0.013 ± 0.007 mGy/MBq) and kidney (mean ± SD: 0.036 ± 0.009 mGy/MBq), were acceptable, compared to other PET diagnostics.

Radiation dosimetry of [^67^Cu]Cu-SAR-BBN was extrapolated from activity distributions of [^64^Cu]Cu-SAR-BBN assuming identical biological clearance for both tracers. Estimated absorbed doses for [^67^Cu]-SAR-BBN are shown in Table 4. Blood data showed rapid clearance of blood pool activity and relatively rapid clearance of renal and hepatic activity.

### 2.4. [^64^Cu]Cu-SAR-BBN and FDG PET Per Patient Analysis

**Figure 1:**
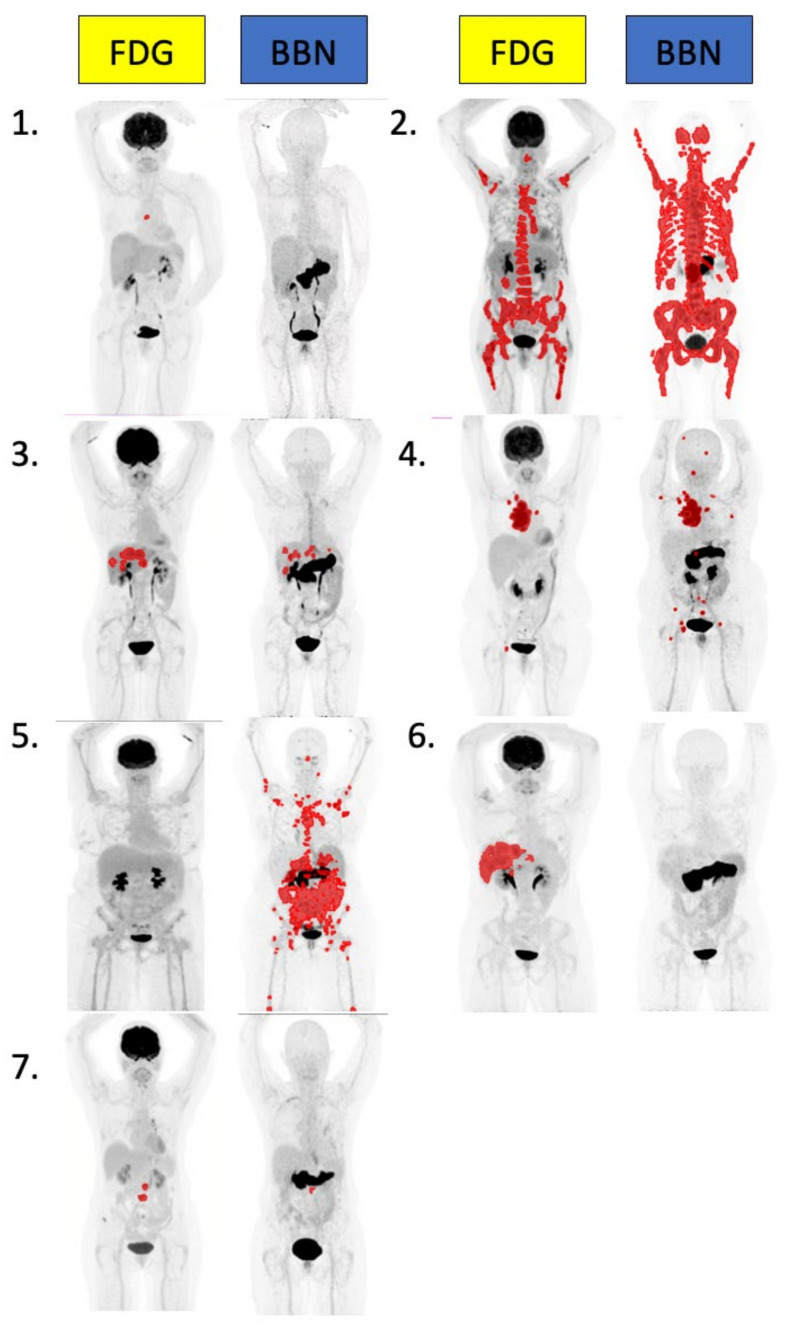
Representative MIPs of total tumor volume quantitation from all 7 patients using MIM software using a threshold of SUVmax >3. BBN quantitation performed on 1 h post-injection acquisition.

Five of seven patients underwent imaging at all three time points, with one of seven missing a 24 h [^64^Cu]Cu-SAR-BBN scan (Patient 3), and one of seven the 3 h [^64^Cu]Cu-SAR-BBN scan (Patient 6). Per patient [^64^Cu]Cu-SAR-BBN and FDG quantitation findings are detailed in Table 5. Maximal intensity projections of FDG and 1 h BBN PET-CTs from all seven patients are shown in Figure 1. All seven patients were positive on conventional imaging (six on FDG-PET, seven on CT and six on bone scan). Five of seven patients had uptake of [^64^Cu]Cu-SAR-BBN consistent with disease. Both [^64^Cu]Cu-SAR-BBN-negative patients had disease identified on FDG. Conversely, one patient was [^64^Cu]Cu-SAR-BBN positive/FDG negative.

### 2.5. [^64^Cu]Cu-SAR-BBN Negative

**Figure 2:**
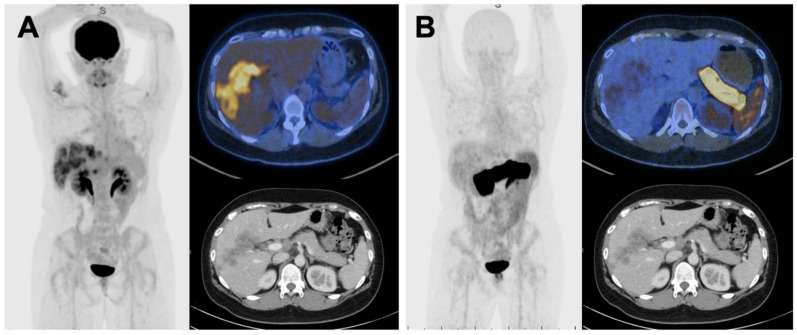
MIP and axial slices of [^18^F]FDG PET-CT (**A**) compared to 1 h [^64^Cu]Cu-SAR-BBN (**B**) in Patient 6. Diagnostic CT shows hypoenhancing liver metastases, confirmed by biopsy. These lesions were FDG positive, BBN negative.

Patient 1 and Patient 6 were negative on [^64^Cu]Cu-SAR-BBN. For Patient 1, liver metastases were negative on both FDG and [^64^Cu]Cu-SAR-BBN PET, but positive on diagnostic CT. The only FDG-PET-positive lesion in this patient was a single lytic bony metastasis. For Patient 6, both liver and nodal metastases were FDG positive, but [^64^Cu]Cu-SAR-BBN negative (Figure 2).

### 2.6. FDG Negative, [^64^Cu]Cu-SAR-BBN Positive

**Figure 3:**
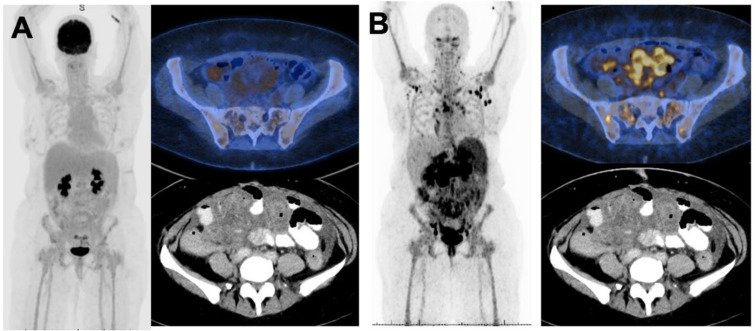
MIP and axial slices from for [^18^F]FDG PET-CT (**A**) compared to 1 h [^64^Cu]Cu-SAR-BBN (**B**) in Patient 5 shows bony, nodal and bowel serosal disease was BBN avid but FDG negative.

Patient 5 was negative on FDG-PET but positive on [^64^Cu]Cu-SAR-BBN-PET (Figure 3). Patient 5 had the lobular carcinoma subtype with large-volume bony, nodal and omental/peritoneal disease, demonstrating high intensity on [^64^Cu]Cu-SAR-BBN (SUV max 19). Omental disease was visible on diagnostic CT, although lymph nodal and bone involvement were not identified on conventional imaging.

### 2.7. [^64^Cu]Cu-SAR-BBN and FDG PET Quantitative Analysis

Individual patient 1 h [^64^Cu]Cu-SAR-BBN and FDG PET quantitative analysis are detailed in Table 5 and combined patient findings detailed in Table 6. [^64^Cu]Cu-SAR-BBN detected a higher mean total tumour volume (TTV) (413 vs. 164 mL) and mean SUVmax (SUVmax 11 vs. 9) across the seven patients combined compared to FDG. SUVmean across the total seven patients did not significantly differ between [^64^Cu]Cu-SAR-BBN and FDG (mean SUVmean 4 vs. 4). Overall, [^64^Cu]Cu-SAR-BBN identified a higher mean number of lesions compared to FDG (49 on BBN vs. 9 on FDG).

In patients positive on both [^64^Cu]Cu-SAR-BBN and FDG PET, [^64^Cu]Cu-SAR-BBN detected a higher mean SUVmax (SUVmax 15 vs. 12) and mean TTV (565 mL vs. 154 mL).

### 2.8. Classical Lobular Subtype

In the classical lobular subtype (two of seven), [^64^Cu]Cu-SAR-BBN-PET was highly avid compared to FDG PET (SUV max 20 vs. 11 (patient 2), and 19 vs. <3 (patient 5)) with a higher tumour volume compared to FDG PET (2034 vs. 504 mL, and 634 vs. 0 mL, respectively).

### 2.9. Biopsy of Metastatic Lesions and Receptor Status

GRPR receptor staining was performed on four metastatic biopsy specimens in four of seven patients (Table 7). GRPR staining was positive in one of four cases. This positive case was a sternal/chest wall lesion (patient 4), intensely avid on both [^64^Cu]Cu-SAR-BBN-PET and FDG PET performed 7 weeks following biopsy.

The other three cases were negative on GRPR staining, including a lesion positive on ^64^Cu-SAR-BBN-PET. One lesion was negative on both ^64^Cu-SAR-BBN-PET and GRPR receptor staining.

## 3. Discussion

We reported the first in-human trial evaluating [^64^Cu]Cu-SAR-BBN as a potential theranostic tool in patients with ER/PR+ HER2-negative metastatic breast cancer. In addition, we describe the first in-human comparison of GRPR-targeted PET with current the standard of imaging, inclusive of FDG PET, in the same subset of patients.

The heterogeneity of breast cancer, both between and within individual patients, poses unique challenges in molecular imaging, making a “one size fits all” targeted approach not currently feasible. As a result, novel targeted radiotracers have been investigated, including [^18^F]fluoro-17ß-estradiol (FES) targeting estrogen receptors, [^64^Cu]Cu-DOTA-transtuzumab targeting HER 2 and [^68^Ga]Ga-PSMA targeting endothelial expression of PSMA [15]. Targeting GRPR is of particular interest, as Morgat et al. observed discordant binding of FDG and GRPR on breast cancer samples, where binding of [^68^Ga]Ga-RM2, a GRPR antagonist, was significantly higher in samples with low FDG binding [16]. This suggests GRPR PET may be complimentary to FDG PET in evaluating ER-positive breast cancer. In our trial, the fact that whilst there were patients negative on FDG or [^64^Cu]Cu-SAR-BBN PET alone, but no patient was negative on both, supports this proposal.

[^64^Cu]Cu-SAR-BBN PET was not superior to FDG in all ER/PR-positive breast cancer patients, but was particularly useful in the lobular carcinoma subtype (ILC). Lower intensity of ILC on FDG PET compared to invasive ductal (IDC) subtypes has been reported previously [17,18,19,20]. This has been attributed to ILC’s infiltrative pattern, reduced cell density, low GLUT1 expression and decreased cell proliferation rate [17]. In contrast, high GRPR expression within ILC specimens was shown to be not inferior to IDC (80.7% vs. 74.6%, respectively) [5]. This is supported by our study, as both ILC patients had greater intensity and tumour volume on [^64^Cu]Cu-SAR-BBN compared to FDG PET.

Only small numbers of ILC patients evaluated with PET agents targeting GRPR (GRPR PET) have been previously described.

Zang et al. and Stoykow et al. reported one of one and three of three ILC patients positive on GRPR PET in the setting of primary staging [21,22]. Zhang et al. reported two ILC cases; however, this was in the setting of a PET heterodimer targeting both GRPR and αvβ3 receptors [23]. Of note, Michalski et al. reported two of two ER-positive metastatic ILC patients negative on GRPR PET. [24] Both patients reported current endocrine therapy for a duration of at least 18 months and one of two patients subsequently had loss of ER expression on examination of metastatic breast cancer cells. In our study, neither patient with ILC was on endocrine therapy at the time of imaging. Loss of ER expression following treatment has been previously described and high GRPR expression is reported in only 12% of ER-negative breast cancer specimens [5].

Two patients in this cohort had negative [^64^Cu]Cu-SAR BBN PET findings. Whilst biopsy specimens for these patients were not assessed via GRPR staining, approximately 17% of ER-positive tumors have previously been shown to have low GRPR expression [5]. Further work in larger trials is required to better define the frequency of negative [^64^Cu]Cu-SAR BBN PET findings in the ER/PR-positive breast cancer population to determine its value as a diagnostic and staging tool.

Targeted radionuclide therapy involves linking a radioactive atom to a targeting molecule and delivering ionizing radiation directly to cancer cells or their microenvironment [25]. Recent investigations into GRPR-targeted radionuclide therapy in the setting of metastatic prostate cancer have been promising. [^177^Lu]Lu-RM2 was well tolerated in a dosimetry study amongst prostate cancer patients with high tumor doses with rapid clearance from normal organs [26]. [^177^Lu]Lu-NeoBOMB1 in animal models demonstrated excellent tumor uptake in prostate cancer cells and favourable pharmacokinetics [27]. [^68^Ga]Ga-NeoBOMB1 has shown similar characteristics in breast cancer animal models and the theranostic pair of [^68^Ga]Ga/[^177^Lu]Lu-NeoBOMB1 is under active investigation in the treatment of breast cancer [28]. Our study highlights the potential of chemically identical radioisotopes (^64^Cu and ^67^Cu). These two radioisotopes have identical in-vivo characteristics, theoretically enabling [^64^Cu]Gu-SAR-BBN PET to precisely assess biodistribution prior to therapy with [^67^Cu]Cu-SAR-BBN. Recently, this theranostic pair of ^64^Cu/^67^Cu-SAR-BBN demonatrated excellent tumor uptake retention with no observed toxicity in prostate cancer animal models [29]. In our study, dosimetry estimates of [^67^Cu]Cu-SAR-BBN were extrapolated by observing the activity distribution on [^64^Cu]Cu-SAR-BBN PET. Dosimetry identified red bone marrow as the likely dose-limiting organ for potential therapy using [^67^Cu]Cu-SAR-BBN in future studies.

A limitation of our study was the small number of participants and single-institution design, meaning the findings from this study are hypothesis generating only. However, this is the first human trial that suggests GRPR-PET may be superior to FDG-PET in patients with ER/PR-positive metastatic breast cancer of the lobular subtype. Further work is needed to confirm these findings in larger studies.

## 4. Materials and Methods

### 4.1. Study Design

This prospective phase 1 study was undertaken at a single Australian Institution (St. Vincent’s Hospital, Sydney, Australia). The study protocol was approved by the St. Vincent’s Hospital institutional review board (HREC/2019/SVH/ETH12700), and patients provided informed and written consent. The study was registered with ANZCTR (ACTRN12619001383156).

### 4.2. Patient Enrolment

Women were considered eligible for the study if they had biopsy-proven metastatic ER+/PR+/HER2- breast cancer requiring staging. Women were excluded if they had an ECOG status higher than 2, or an active second malignancy. Clinical information including whether the patient was de novo or metastatic at diagnosis, history of prior treatments, and both diagnostic and metastatic histopathology including receptor status were all documented. All patients imaged underwent [^64^Cu]Cu-SAR-BBN PET-CT, diagnostic CT of the chest, abdomen and pelvis, [^99^Tc]Tc-MDP bone scan and [^18^F]FDG PET-CT within a 3-week period. 

### 4.3. [^64^Cu]Cu-SAR-BBN Production and Quality Control

[^64^Cu]Cu-SAR-BBN was prepared by the South Australian Health & Medical Research Institute SAHMRI (Adelaide, Australia) using a modified version of a previously described method [11]. [^64^Cu]CuCl_2_ (1000–2100 MBq, 500 μL, 0.05 M HCl; SAHMRI) was added to SAR-BBN (Auspep Pty Ltd., Melbourne, Australia) (60 μg) in 5 mL of a solution of sodium phosphate buffer (0.1 M, pH 6.5–7.0) containing sodium gentisate (4 mg). The reaction mixture was incubated for 25 min at room temperature, after which the reaction mixture was filtered through a 0.22 μM filter into a sterile product vial. The reaction was quenched by addition of 10 mL of a 5% aqueous ethanol solution containing sodium ascorbate (750 mg) via the 0.22 μM filter into the sterile product vial. [^64^Cu]Cu-SAR-BBN was produced in an average 84% radiochemical yield with more than 95% radiochemical purity. [^64^Cu]Cu-SAR-BBN was injected within 24 h of production in all patients.

### 4.4. [^64^Cu]Cu-SAR-BBN Imaging

Patients were injected with 200 MBq (median dose 205 MBq) of intravenous [^64^Cu]Cu-SAR-BBN. PET-CT imaging was conducted at 1, 3 and 24 h post injection. [^64^Cu]Cu-SAR-BBN PET-CT was performed on Siemens Biograph Vision 600–64 PET-CT scanner at 90 s per frame. Whole-body [^64^Cu]Cu-SAR-BBN PET (skull vertex to mid-thigh) was preceded by a non-contrast low-dose CT scan using the following CT parameters: slice thickness of 2 mm, with 2 mm increment, soft-tissue reconstruction kernel, 120 kV and 50 mAs, pitch of 0.8 and a 440 matrix. Emission data were corrected for randoms, scatter and decay using the Siemens body.xml reconstruction protocol.

### 4.5. FDG Protocol

FDG PET-CT was performed as per clinical standard of care using the same PET-CT scanner and parameters as for [^64^Cu]Cu-SAR-BBN. Following a six-hour fast, patients were injected with [^18^F]Fluorodeoxyglucose intravenously (3.5 MBq/kg, median dose 227 MBq). Blood sugar levels (BSL) were less than 13 mmol/L (median BSL 5.1 mmol/L) prior to injection. Subsequently, 60 min after injection (median time 62 min), non-contrast low-dose CT scan was obtained followed by whole-body PET scan.

### 4.6. Bone Scan and Diagnostic CT

Bone scan was performed using a clinical protocol using a dual-head gamma camera (Discovery 670) equipped with a low-energy general-purpose collimator. Patients were injected with 800 MBq of [^99m^Tc]Tc-MDP with whole-body imaging at 3 h. Diagnostic CT scan of the chest, abdomen and pelvis with intravenous contrast was performed as per standard of care.

### 4.7. Image Interpretation

All PET-CT images were reported using the SIEMENS FUSION Viewer (Syngo). All studies were interpreted by credentialed nuclear medicine physicians with experience in reporting PET-CT images. Data for all scans were analysed both visually and quantitatively. Identification of positive sites of disease on PET was determined clinically and quantitatively. Quantitative analysis was undertaken to calculate total tumour burden as well as overall tumour versus individual lesion SUV max and SUV mean data (MIM Software, Cleveland) using an SUV max minimum value of 3 and volume 0.2 mL. Bone and CT scans were interpreted by the same nuclear medicine physicians using RECIST criteria.

### 4.8. Safety Assessment

Haematological, biochemical and coagulation assays were collected at baseline. Vital signs and safety physical examinations were conducted at baseline, and at 1, 3 and 24 h post injection. Electrocardiogram (ECG) was performed at 1 h pre and 1 h post injection.

### 4.9. Dosimetry

Radiation dosimetry of [^64^Cu]Cu-SAR-BBN was determined using standard methods established by the Committee on Medical Internal Radiation Dose (MIRD) and implemented inside the 3D-RD-S software package. Time–activity curves were generated from volumes-of-interest defined for source organs within the images and estimated absorbed dose to all International Commission on Radiological Protection target tissues were calculated using the MIRD S-value methodology. Once the absorbed dose for all target tissue was obtained, the effective dose was calculated as the average absorbed dose over all tissues with each tissue dose weighted by the corresponding tissue-weighting factor. Blood tracer clearance was determined radio-metrically as described below.

### 4.10. Blood Activity

In order to investigate radiopharmaceutical clearance, 2 × 1 mL blood samples were taken at baseline, 1 h, 3 h and 24 h post injection. These were counted on a Perkin Elmer Wizard2 gamma counter together with 5 sets of 2 × 1 mL standards of approximately 0.6, 1.0, 2.5, 5.0 and 8.0 kBq/mL. The standards were prepared within 1 h from the collection of the blood sample at 3 h post acquisition. The counts to activity conversion factor was estimated using all standards and blood activity concentration of each sample was decay corrected to the time of injection. All patient data were fitted to a bi-exponential function using R (R CORE TEAM 2021).

### 4.11. Metastatic Biopsy and GRPR Staining

Histopathology records for metastatic biopsy were sourced. Available tissue samples underwent GRPR receptor status staining. Tissue was formalin fixed and paraffin embedded. Mild antigen retrieval was performed using Cell Conditioning for 36 min at 95 °C. Polyclonal antibody TA316872 (OriGene) to GRPR was used at a dilution of 1/200 and incubated at 36 °C for 32 min. Ultraview was used for detection. Slides were scored by a consultant pathologist with cytoplasmic staining as positive.

## 5. Conclusions

[^64^Cu]Cu-SAR-Bombesin appears safe and may have a diagnostic and theranostic utility in subtypes of metastatic ER+/PR+/HER2- breast cancer patients, especially the lobular subtype. Further investigation is warranted.

## Figures and Tables

**Table 1:** Patient characteristics.

Median Age (Years)	60 (38–81)
**Median time from diagnosis (years)**	3 (0–29)
**Type of primary cancer**	
Invasive Carcinoma NOS	5
Invasive lobular carcinoma	2
**Stage (at diagnosis)**	
M0	5
M1	1
Unknown	1
**Number of lines of prior therapy**	
0	1
1	3
2 or more	3
**Most immediate prior therapy**	
Chemotherapy	2
Endocrine therapy	0
Targeted therapy (OTHERS)	4

**Table 2:** Patient characteristics.

**Pt**	**Histological Subtype**	**ER/PR (%) Primary**	**Metastatic Biopsy** site	**ER/PR (%) Metastatic**	**Immediate Prior therapy**
**1**	Invasive ductal	90/80	Liver	90/−	Letrozole/ Palbociclib
**2**	Classical lobular	n/a	Liver	95/70	Vinorelbine
**3**	Invasive ductal	95/95	Liver	+/+	Fulvestrant/ Palbociclib
4	Invasive ductal	+/+	Chest wall	95/95	Letrozole
**5**	Classical lobular	n/a	Skin	95/−	−
**6**	Invasive ductal	80/−	Liver	90/−	Carboplatin/ Gemcitabine
**7**	Invasive ductal	62/53	Pleura	100/5	Letrozole/Ribociclib

“+” indicates positive and “−“ indicate negative immunohistochemistry receptor staining.

**Table 3:** Effective dose (mSv/MBq) for [^64^Cu]Cu-SAR-BBN. Mean and standard deviation (SD) are shown across all subjects.

Patient	1	2	3	4	5	6	7	Mean ± SD
**Effective Dose** **(mSv/MBq)**	0.0116	0.0124	0.0080	0.0069	0.0084	0.0111	0.0083	0.0095 ± 0.0021

**Table 4:** Estimated absorbed doses (Gy) at various administered activities of [^67^Cu]Cu-SAR-BBN, shown as mean ± standard deviation. Dose limits [12,13,14].

Target Tissues	Estimated Absorbed Dose in Gy					Dose Limits (Gy)
GBq/Patient	10	20	40	60	87	
**Pancreas**	3.0 ± 1.2	6.1 ± 2.5	12.1 ± 5.0	18.2 ± 7.5	26.3 ± 10.8	-
**Kidneys**	0.7 ± 0.3	1.4 ± 0.5	2.8 ± 1.0	4.2 ± 1.5	6.0 ± 2.2	23
**Alveolar-interstitial**	0.4 ± 0.1	0.8 ± 0.2	1.5 ± 0.4	2.3 ± 0.6	3.3 ± 0.8	20
**Liver**	0.3 ± 0.1	0.6 ± 0.2	1.2 ± 0.4	1.8 ± 0.6	2.6 ± 0.9	30
**Red (active) marrow**	0.2 ± 0.1	0.5 ± 0.3	0.9 ± 0.6	1.4 ± 0.9	2.0 ± 1.3	2
**Spleen**	0.3 ± 0.1	0.5 ± 0.1	1.0 ± 0.3	1.5 ± 0.4	2.2 ± 0.5	
**Bronchioles secretary cells**	0.2 ± 0.0	0.4 ± 0.1	0.8 ± 0.2	1.1 ± 0.3	1.7 ± 0.4	
**Heart wall**	0.2 ± 0.0	0.4 ± 0.1	0.7 ± 0.2	1.1 ± 0.3	1.5 ± 0.4	26

**Table 5:** Individual patient histological and whole-body quantitation data.

Pt	Histological Subtype	FDG		BBN (1 h)	
		SUV Max	TV	SUV Max	TV
**1**	Invasive ductal	7	2	<3	0
**2**	Classical lobular	11	504	20	2033
**3**	Invasive ductal	7	91	6	13
**4**	Invasive ductal	20	168	27	209
**5**	Classical lobular	<3	0	19	634
**6**	Invasive ductal	7	381	<3	0
**7**	Invasive ductal	8	8	5	3

**Table 6:** Mean total tumor volume (TTV), SUV max, SUV mean and number of lesions by quantitative combined patient BBN vs. FDG analysis.

	BBN (1 h)	FDG
**TTV**	413 (751)	164 (202)
**SUV Max**	11 (11)	9 (6)
**SUV Mean**	4 (3)	4 (3)
**No. of lesions**	49 (74)	9 (12)

**Table 7:** GRPR staining of biopsies of metastatic sites.

Patient	Metastatic Site	GRPR Staining	FDG SUV Max	BBN 1 h SUV Max
**2**	Liver	Negative	4.4	11.5
**3**	Liver	Negative	4.6	-
**4**	Sternum	Positive	4.4	11.5
**5**	Skin Lesion *	Negative	n/a	n/a

* Lesion completely excised prior to FDG and BBN PET.

## Data Availability

Data is contained within the article.

## References

[1] National Breast Cancer Foundation Breast Cancer Stats. https://nbcf.org.au/about-breast-cancer/breast-cancer-stats/.

[2] Choi Y.J., Jeong Y.H., Kim H.J., Lee J.H., Cho A., Yun M., Lee J.D., Kang W.J. (2012). Correlation between hormonal receptor status/human epidermal growth factor receptor 2 overexpression and 18F-FDG uptake in patients with breast cancer. J. Nucl. Med..

[3] Yoon H.J., Kang K.W., Chun I.K., Cho N., Im S.A., Jeong S., Lee S., Jung K.C., Jeong J.M., Moon W.K. (2014). Correlation of breast cancer subtypes, based on estrogen receptor, progesterone receptor, and HER2, with functional imaging parameters from ⁶⁸Ga-RGD PET/CT and ¹⁸F-FDG PET/CT. Eur. J. Nucl. Med. Mol. Imaging.

[4] Basu S., Chen W., Tchou J., Mavi A., Cermik T., Czerniecki B., Schnall M., Alavi A. (2008). Comparison of triple-negative and estrogen receptor-positive/progesterone receptor-positive/HER2-negative breast carcinoma using quantitative fluorine-18 fluorodeoxyglucose/positron emission tomography imaging parameters: A potentially useful method for disease characterization. Cancer.

[5] Morgat C., MacGrogan G., Brouste V., Vélasco V., Sevenet N., Bonnefoi H., Fernandez P., Debled M., Hindie E. (2017). Expression of Gastrin-Releasing Peptide Receptor in Breast Cancer and Its Association with Pathologic, Biologic, and Clinical Parameters: A Study of 1,432 Primary Tumors. J. Nucl. Med..

[6] Parry J.J., Andrews R., Rogers B.E. (2007). MicroPET imaging of breast cancer using radiolabeled bombesin analogs targeting the gastrin-releasing peptide receptor. Breast Cancer Res. Treat..

[7] Gugger M., Reubi J.C. (1999). Gastrin-releasing peptide receptors in non-neoplastic and neoplastic human breast. Am. J. Pathol..

[8] Scopinaro F., Varvarigou A., Ussof W., De Vincentis G., Archimandritis S., Evangelatos G., Corleto V., Capoccetti F., Massa R., Remediani S. (2002). Breast cancer takes up 99mTc bombesin. A preliminary report. Tumori J..

[9] Van de Wiele C., Phonteyne P., Pauwels P., Goethals I., Van den Broecke R., Cocquyt V., Dierckx R.A. (2008). Gastrin-Releasing Peptide Receptor Imaging in Human Breast Carcinoma Versus Immunohistochemistry. J. Nucl. Med..

[10] Huynh T.T., van Dam E.M., Sreekumar S., Mpoy C., Blyth B.J., Muntz F., Harris M.J., Rogers B.E. (2022). Copper-67-Labeled Bombesin Peptide for Targeted Radionuclide Therapy of Prostate Cancer. Pharmaceuticals.

[11] Gourni E., Del Pozzo L., Kheirallah E., Smerling C., Waser B., Reubi J.C., Paterson B.M., Donnelly P.S., Meyer P.T., Maecke H.R. (2015). Copper-64 Labeled Macrobicyclic Sarcophagine Coupled to a GRP Receptor Antagonist Shows Great Promise for PET Imaging of Prostate Cancer. Mol. Pharm..

[12] O’ Donoghue J.A., Baidoo N., Deland D., Welt S., Divgi C.R., Sgouros G. (2002). Hematologic Toxicity in Radioimmunotherapy: Dose-Response Relationships for I-131 Labeled Antibody Therapy. Cancer Biother. Radiopharm..

[13] Dawson L.A., Kavanagh B.D., Paulino A.C., Das S.K., Miften M., Li X.A., Pan C., Haken R.K.T., Schultheiss T.E. (2010). Radiation-associated kidney injury. Int. J. Radiat. Oncol. Biol. Phys..

[14] Marks L.B., Yorke E.D., Jackson A., Ten Haken R.K., Constine L.S., Eisbruch A., Bentzen S.M., Nam J., Deasy J.O. (2010). Use of normal tissue complication probability models in the clinic. Int. J. Radiat. Oncol. Biol. Phys..

[15] Ming Y., Wu N., Qian T., Li X., Wan D.Q., Li C., Li Y., Wu Z., Wang X., Wu N. (2020). Progress and Future Trends in PET/CT and PET/MRI Molecular Imaging Approaches for Breast Cancer. Front. Oncol..

[16] Morgat C., Schollhammer R., Macgrogan G., Barthe N., Vélasco V., Vimont D., Cazeau A.-L., Fernandez P., Hindié E. (2019). Comparison of the binding of the gastrin-releasing peptide receptor (GRP-R) antagonist 68Ga-RM2 and 18F-FDG in breast cancer samples. PLoS ONE.

[17] Fujii T., Yajima R., Kurozumi S., Higuchi T., Obayashi S., Tokiniwa H., Nagaoka R., Takata D., Horiguchi J., Kuwano H. (2016). Clinical Significance of 18F-FDG-PET in Invasive Lobular Carcinoma. Anticancer Res..

[18] Groheux D., Cochet A., Humbert O., Alberini J.L., Hindié E., Mankoff D. (2016). ¹⁸F-FDG PET/CT for Staging and Restaging of Breast Cancer. J. Nucl. Med..

[19] Hogan M.P., Goldman D.A., Dashevsky B., Riedl C.C., Gönen M., Osborne J.R., Jochelson M., Hudis C., Morrow M., Ulaner G.A. (2015). Comparison of 18F-FDG PET/CT for Systemic Staging of Newly Diagnosed Invasive Lobular Carcinoma Versus Invasive Ductal Carcinoma. J. Nucl. Med..

[20] Jung N.Y., Kim S.H., Kim S.H., Seo Y.Y., Oh J.K., Choi H.S., You W.J. (2015). Effectiveness of Breast MRI and (18)F-FDG PET/CT for the Preoperative Staging of Invasive Lobular Carcinoma versus Ductal Carcinoma. J. Breast Cancer.

[21] Zang J., Mao F., Wang H., Zhang J., Liu Q., Peng L., Li L., Lang L., Chen X., Zhu Z. (2018). 68Ga-NOTA-RM26 PET/CT in the Evaluation of Breast Cancer: A Pilot Prospective Study. Clin. Nucl. Med..

[22] Stoykow C., Erbes T., Maecke H.R., Bulla S., Bartholomä M., Mayer S., Drendel V., Bronsert P., Werner M., Meyer P.T. (2016). Gastrin-releasing Peptide Receptor Imaging in Breast Cancer Using the Receptor Antagonist (68)Ga-RM2 And PET. Theranostics.

[23] Zhang J., Mao F., Niu G., Peng L., Lang L., Li F., Chen X., Ying H., Wu H., Pan B. (2018). (68)Ga-BBN-RGD PET/CT for GRPR and Integrin α(v)β(3) Imaging in Patients with Breast Cancer. Theranostics.

[24] Michalski K., Kemna L., Asberger J., Grosu A.L., Meyer P.T., Ruf J., Sprave T. (2021). Gastrin-Releasing Peptide Receptor Antagonist [(68)Ga]RM2 PET/CT for Staging of Pre-Treated, Metastasized Breast Cancer. Cancers.

[25] Sgouros G., Bodei L., McDevitt M.R., Nedrow J.R. (2020). Radiopharmaceutical therapy in cancer: Clinical advances and challenges. Nat. Rev. Drug Discov..

[26] Kurth J., Krause B.J., Schwarzenböck S.M., Bergner C., Hakenberg O.W., Heuschkel M. (2020). First-in-human dosimetry of gastrin-releasing peptide receptor antagonist [(177)Lu]Lu-RM2: A radiopharmaceutical for the treatment of metastatic castration-resistant prostate cancer. Eur. J. Nucl. Med. Mol. Imaging.

[27] Dalm S.U., Bakker I.L., de Blois E., Doeswijk G.N., Konijnenberg M.W., Orlandi F., Barbato D., Tedesco M., Maina T., de Jong M. (2017). 68Ga/177Lu-NeoBOMB1, a Novel Radiolabeled GRPR Antagonist for Theranostic Use in Oncology. J. Nucl. Med..

[28] Kaloudi A., Lymperis E., Giarika A., Dalm S., Orlandi F., Barbato D., Tedesco M., Maina T., De Jong M., Nock B.A. (2017). NeoBOMB1, a GRPR-Antagonist for Breast Cancer Theragnostics: First Results of a Preclinical Study with [(67)Ga]NeoBOMB1 in T-47D Cells and Tumor-Bearing Mice. Molecules.

[29] Huynh T., Van Dam E., Houston Z., McInnes L., Mpoy C., Harris M., Thurecht K., Donnelly P., Rogers B. (2021). A Cu-64/Cu-67 Bombesin ligand as a theranostic for cancer. J. Nucl. Med..

